# Pectin as a Versatile Biomaterial: Structure, Green Sourcing, and Emerging Applications in Pharmaceutics and Biomedicine

**DOI:** 10.3390/ijms27083518

**Published:** 2026-04-15

**Authors:** Irina-Alexandra Dumitrescu, Cristina-Elena Dinu-Pîrvu, Mihaela Violeta Ghica, Valentina Anuța, Răzvan Mihai Prisada, Lăcrămioara Popa

**Affiliations:** 1 Department of Physical and Colloidal Chemistry, Faculty of Pharmacy, “Carol Davila” University of Medicine and Pharmacy, 6 Traian Vuia Str., 020956 Bucharest, Romania; irina-alexandra.rotaru@drd.umfcd.ro (I.-A.D.); cristina.dinu@umfcd.ro (C.-E.D.-P.); valentina.anuta@umfcd.ro (V.A.); razvan.prisada@umfcd.ro (R.M.P.); lacramioara.popa@umfcd.ro (L.P.); 2 Innovative Therapeutic Structures Research and Development Center (InnoTher), “Carol Davila” University of Medicine and Pharmacy, 6 Traian Vuia Str., 020956 Bucharest, Romania

**Keywords:** hydrocolloid, pectin, “green” methods, eco-pharmacy

## Abstract

Hydrocolloids are an extremely diverse and valuable group of materials, with various sources, properties and applications in many industries. Increasingly, naturally sourced colloids have gained the interest of the scientific world for their bio-availability, eco-friendliness and bio-degradability. This, coupled with emerging “green” extraction methods and modifying techniques, opens a wide range of uses. Pectin is a well-known, natural and abundant biomaterial, a heterogeneous anionic polysaccharide with vast applications in the food and pharmaceutical industries. Traditionally used in the food sector as a gelling agent and thickener, it is considered safe for human consumption. Pectin has found new applications in the pharmaceutical and medical worlds due to its complex structure, and it provides variety in its properties. This paper brings together information about this polysaccharide’s genuine usefulness in the context of growing interest for naturally sourced polymers, the reduction in wasteful industrial practices and environmental protection.

## 1. Introduction

Hydrocolloids are a well-known and extensively studied group of materials. Synthetic and natural, they have been described and classified in detail by many. Hydrocolloid-based hydrogels are polymeric 3D structures that are capable of swelling with water or biological fluids. Their structure makes them very useful in many areas of application [1].

In recent years, focus has shifted from synthetic polymers to naturally sourced ones in an attempt on the part of the scientific community to align with a more environmentally friendly approach to science. The use of polymers for obtaining hydrogels has been studied for the last 60 years, starting with the research of Drahoslav Lim and Otto Wichtarle of the Institute of Chemical Technology in Prague in 1960. They designed hydrogels based on poly (2-hydroxyethyl methacrylate) and inferred the possibility of using them to manufacture contact lenses and arteries. They created the first biomaterial that could be used for humans for the manufacturing of contact lenses [2].

Previously, the use of the term “hydrogel” had been used by Dutch chemist Jakob Maarten van Bemmelen, in 1894. His work referred to the production of hydrogel from inorganic salts [3]. Hydrogel’s ability to retain a massive amount of fluid/water without dissolution is its basic and most important characteristic. While maintaining their structure, hydrogels also display flexibility, biocompatibility and stimuli-responsiveness [4].

In the family of naturally sourced hydrocolloids, pectin has an important role to play. Initially used in the food industry as a gelling and thickening agent, pectin has found new applications in the pharmaceutical and medical industries.

Pectin is a complex natural polysaccharide primarily extracted from citrus peels, apples or sugar beet, traditionally used in the food industry as a gelling agent (E440i). In recent decades, due to its high biocompatibility, biodegradability, and low toxicity, pectin has attracted increasing interest as a biomaterial for modern biomedical applications [5]. Its chemical structure—based on partially methylated D-galacturonic acid chains branched with rhamnogalacturonan regions—provides multiple sites for chemical modification, allowing fine-tuning of its physicochemical and biological properties [6]. Pectins can form gels in the presence of ions (e.g., Ca^2+^ for low-methoxy pectins) or through hydrogen bonding in acidic sugar media (high-methoxy pectins), making them versatile as pharmaceutical excipients. Moreover, pectin exhibits intrinsic bioactive properties (immunomodulatory, anti-inflammatory, antioxidant, antitumor, etc.) that can enhance therapeutic effects [7].

More recently, pectins have been explored as part of innovative drug delivery systems in combination with other polymers [8,9,10,11]. This abundant, naturally sourced polysaccharide has a complex structure that can be modified to suit a particular purpose [12,13,14,15]. Pectin also has many intrinsic protective properties that add to its value in the pharmaceutical industry [16]. As the study on the potential of pectin continues, it has been proposed as a novel stabilizing agent in Pickering emulsions [17].

The importance of being conscientious regarding the impact of daily activities on the environment is at the basis of the research that combines the needs of society with a certain degree of care towards nature. In that sense, the idea of sustainability is very easy to understand, but at times hard to put into practice. The purpose of eco-pharmacy is the development of novel techniques and methods of extractions, applications and disposal that are safe for the environment.

This synthesis reviews state-of-the-art applications of pectin in various biomedical fields, regulatory aspects and safety profile, main derivatives used and current trends in pectin research.

## 2. A General Overview of Hydrocolloids

Research is being done in several fields of science on the development of better performing hydrocolloid-based hydrogels for agriculture [18], the food industry [19], biomaterials [20,21], wound dressing [22], drug delivery [1,23], wastewater treatment [24] and many others.

Although in the beginning, the research of hydrocolloid-based hydrogels started with synthetic polymers, today a lot of work is put into natural-sourced monomers and polymers [25,26]. Natural-sourced monomers and polymers have several advantages over the synthetic ones, such as biocompatibility, biodegradability and being environmentally friendly.

Hydrogels have the ability to imbibe a large amount of water, due to the hydrophilic polymer skeleton within their structure, while maintaining said structure. This property is referred to as swelling. The swelling capacity of hydrogels can be altered to suit the needs of particular uses. For instance, in the case of biomedical applications, such as tissue engineering and bioelectronics, a low swelling hydrogel is required to preserve structure and rigidity [27]. The amount of fluid a particular hydrogel may absorb is related to the hydrophilic molecules in its structure, mainly -OH, -CONH, -CONH_2_ and -SO_3_H [28].

Mesh size is an important and measurable property of hydrocolloid-based hydrogels, and it refers to the size of the pores between the polymeric chains. It decreases with the increase in cross-linking density [29].

Not all hydrocolloids have the ability to form gels, despite their viscosity-increasing properties. Most known gel-forming hydrocolloid natural sourced materials are agar, alginate, gelatine, pectin, gellan gum, starch, methylcellulose, chitosan, carrageenan [26]. There are several criteria by which a classification of hydrocolloid-based hydrogels might be made: source of the polymers, configuration, type of cross-linking, physical appearance, and network electrical charge [30].

The hydrogel network formation mechanism has two stages: (i) The macromolecular polymer chains are linked together into a polydisperse structure that retains its solubility and is named ‘sol’, and (ii) the continuation of the linking process increases the size of the structure and ultimately decreases its solubility, creating what is called a ‘gel’. The transition from ‘sol’ to ‘gel’ is called ‘gelation’, and the moment the gel appears is the ‘gel point’ [31]. Links between the polymer chains differ and therefore their strength. Physical links usually create reversible and weaker structures, while chemical links provide permanent, stronger ones [32].

With regard to the benefits of hydrocolloids in pharmaceutical formulations, several ones from varied sources have found use in the pharmaceutical and medical industries. Firstly, synthetic colloids and lately natural ones are employed with the purpose of improving pharmaceutical formulations. As research continues to focus on “green” materials, many well-known hydrocolloids find new avenues for application.

## 3. Pectins: Properties and Sources

### 3.1. Natural Sources of Pectins, Extraction and Purification Methods

Pectin is extremely well represented in the natural world, as this polysaccharide is found in the cell wall of superior plants, mainly in the middle lamella. The cell wall plants mainly comprise three classes of polysaccharides: cellulose, hemicellulose, and pectin. As an anionic carbohydrate, pectin is important in superior plants’ structural stability by providing rigidity to the cell wall and contributing to cell adhesion. Pectins can also be found in different *Streptophyta* sp. and green algae (*Chlorophyta*), members of which have a global distribution in aquatic freshwater and marine habitats [33].

Traditionally, different varieties of citrus and apples are the usual sources of pectin because these provide the highest extraction yields by conventional methods. However, various fruit and vegetable processing by-products have been investigated as alternative pectin sources and finding use for “leftovers” is in keeping with the ideas of a circular economy. A number of these alternative pectin sources are listed in Table 1, showing also a variety of extraction methods and yields. Usually, on an industrial level, pectin is extracted from citrus and apple peels [34,35] and in a smaller amount from sugar beet [36]. However, pectin can be found and extracted from many sources such as apple pomace [37], pineapple peel, watermelon rind [38,39], mango peel [40], banana peel [41], sweet potato peel [42], cocoa husk, okra pods, pumpkin peel and others.

The extraction methods usually involve acidic or basic hydrolysis combined with enzymatic treatment or a chelating agent. Dependent on the type of hydrolysis, two types of pectin might be obtained. If acidic hydrolysis is used, the pectin will be high-methoxylated, whereas with basic hydrolysis, the pectin will be low-methoxylated. After extraction, alcoholic precipitation is used to separate the impurities [43]. This method has a high energy and solvent cost and takes a long time (1–6 h). There are several other methods of extracting pectin that are generally considered “greener”, but the yield is not as satisfactory as the traditional methods [15,44].

Some of the newer methods are enzyme extraction [45], microwave-assisted extraction [14,46,47], ultrasound-assisted extraction [48], subcritical water extraction [49,50], extraction with deep eutectic solvents [15] and extraction with natural deep eutectic solvents [51].

**Table 1:** Alternate sources of pectin and extraction methods.

Emerging Sources of Pectin	Method of Extraction	Yield %	References
Jackfruit peel	Subcritical water extraction,	14.5–30.42	[14,52]
	Acidic extraction		
Mango peel	Acidic extraction;	1.55–21.82	[53,54,55,56]
	Ultrasound extraction assisted citric acid		
Potato pulp	Acidic extraction	14.34	[57]
Pomegranate peel	Citric acid ultrasound extraction	8.5–31.0	[58,59,60]
Banana peel	Acidic extraction	15.89–24.08	[47,61,62]
	Ultrasound extraction		
	Microwave-assisted extraction		
Cocoa pod husk	Acidic extraction	8.00–11.31	[63,64]
	Ultrasound extraction		
	Microwave-assisted extraction		
Sunflower heads	Conventional heating	7.36–14.5	[65,66]
	Ultrasonic extraction, superfine grinding pretreatment		
Soy	Conventional heating	26.00–28.00	[67]
Grape pomace	Microwave-assisted extraction	3.96–11.20	[46]
Okra pods	Hot buffer extraction at pH 6.0	11.00–14.00	[68]
Strawberry	Conventional heating, Ultrasonic extraction, Enzymatic extraction	4.10–9.00	[69]

### 3.2. Chemical Structure of Pectins

Pectin is a naturally occurring heteropolysaccharide that can be found in the cell wall of superior plants. As it is a natural polymer, pectin has a complex structure mostly comprising α-(1-4)-linked D-galacturonic acid units linked on a backbone, with units of 1,2-β-l-rhamnose [70].

Pectic polysaccharides are present in plants and have a structural function, predominantly as a mechanical strengthener and cell adhesion agent [38]. Due to the variability and complexity of its chemical structure, it is appropriate to regard this biomaterial as a group rather than a single compound. This variation in structure is explained by the different plants from which pectin is extracted, as well as the extraction method. Most commonly, pectin structure may consist of homogalacturonan and rhamnogalacturonan I, which are covalently linked, and to a smaller degree, rhamnogalacturonan II and xylogalacturonan [52].

Usually, pectin is constituted of approximately 65% homogalacturonan, about 20–35% rhamnogalacturonan I, and up to 10% rhamnogalacturonan II. Homogalacturonan is built of galacturonic acid units and has a linear structure, with the ability to crystallize, while rhamnogalacturonan I and rhamnogalacturonan II are branched and provide a branched structure also known as “hairy”. Types of pectin with a large percentage of homogalacturonan are used as thickeners and gels, especially in the food industry, but also in the pharmaceutical and cosmetics industries. However, rhamnogalacturonan I -enriched pectic polysaccharides have properties that make them useful in anti-cancer or immunomodulatory therapies, and also as prebiotics [71].

**Figure 1:**
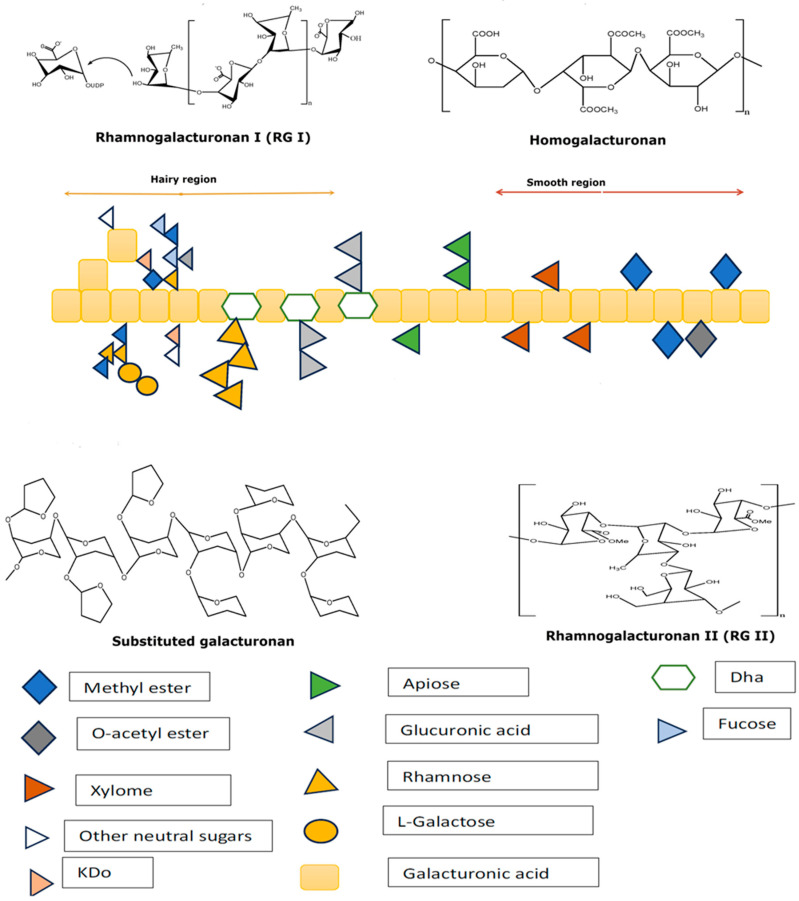
General structure of pectin—adapted from Dambuza et al. (2024) [6].

The complexity of its structure, as shown in Figure 1, explains the variation in pectin’s properties (gelling capacity, rheological behavior, solubility) and the many different uses in the pharmaceutical, food and biomedical industries [72].

### 3.3. Physicochemical Properties of Pectins

The degree of esterification is also known as the degree of methoxylation of pectin, and it refers to the number and distribution of methyl-ester groups (the index of carboxyl groups that can be esterified with methyl groups). It is an extremely important parameter to determine, as it influences pectin’s properties and consequently its possible uses.

High-methoxylated pectin has a degree of esterification of 50% or above and forms gels in a low pH medium (2.5–3.5) in the presence of sucrose or other soluble solids. The 3D network of high-methoxylated pectin gels is formed through connecting zones that are stabilized by hydrogen bonding between carboxyl and secondary alcohol groups, as well as hydrophobic interactions involving methyl esters. The vast majority of pectins found in nature are high-methoxylated [43,73].

Low-methoxylated pectin has a degree of esterification below 50% and forms gels at a wide pH range (between 2 and 6) in the presence of Ca^2+^ ions, by a similar mechanism to alginate’s egg-box model [74]. Pectin has found use in the pharmaceutical and medical world through biological activities [7,75].

### 3.4. Pectin in the Context of Other Natural Polymers

To offer an accurate overview of pectins’ place among other biopolymers, it is appropriate to do a comparison between them and several other materials that have also been used in the development of drug delivery systems.

Pectin occupies a distinct position among natural polymers commonly used in pharmaceutical and biomedical applications, including alginate, chitosan, gelatine, and cellulose derivatives. Like alginate, pectin is an anionic polysaccharide capable of ionic gelation in the presence of divalent cations such as Ca^2+^, following a similar egg-box crosslinking mechanism; however, pectin offers the additional advantage of pH-responsive behavior and colon-specific degradation by microbial enzymes, making it more suitable for targeted gastrointestinal delivery [76]. Chitosan, by contrast, is a cationic polymer with strong mucoadhesive and antimicrobial properties, but its use is limited by poor solubility at physiological pH and concerns regarding its animal origin; pectin complements chitosan well in polyelectrolyte complexes, where the two oppositely charged polymers combine to produce systems with enhanced stability and mucoadhesion [77]. Gelatin, obtained by the hydrolysis of collagen, offers excellent biocompatibility and cell-adhesive properties useful in tissue engineering, but it lacks pectin’s intrinsic bioactive properties—particularly its immunomodulatory and antitumour activities—and degrades rapidly at physiological temperatures without chemical crosslinking [78]. Cellulose derivatives such as hydroxypropyl methylcellulose and carboxymethylcellulose are widely used as thickeners and film formers, but are not degraded by the human body and lack the biological activity of pectin [76]. Overall, pectin’s combination of plant-based origin, tunable gelling behavior, intrinsic bioactivity, colonic degradability, and regulatory acceptance positions it favorably relative to these alternatives, particularly for sustainable and multifunctional biomedical applications.

## 4. Modern Applications of Pectin in Drug Delivery Systems and Emerging Biomaterials

**Figure 2:**
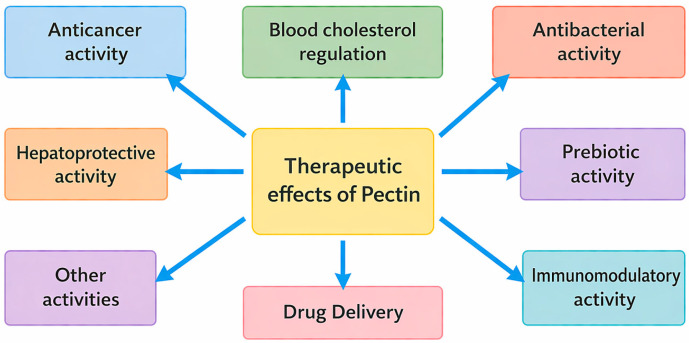
A schematic of inferred therapeutic effects of pectin.

The discovery and first description of pectin as a polysaccharide in 1825 by Henry Braconnot opened many possibilities for obtaining this material. Starting with traditional and pursuing novel sources, exploring various extraction techniques and eventually investigating the modifying possibilities and studying probable applications [79]. Figure 2 gives a summary description of the relevant therapeutic effects of pectin that have been investigated.

Pectin is traditionally used as an excipient in pharmaceutical formulations due to its excellent binding capacity, but as studies progressed, it has been revealed that pectin has intrinsic health benefits, presented in Table 2, both for its potentially preventative activity and for its capacity to ameliorate various afflictions.

This family of heteropolysaccharides, pectins, has been studied with regard to colon and breast cancer. Pectin has the ability to travel the length of the digestive tract intact and ultimately be degraded almost completely in the colon by pectinolytic enzymes produced by the gut microflora [80]. Pectin’s anti-cancerous potential has been explored at length and has not been exhausted as of yet [81,82].

Pectin was shown to be highly effective in inhibiting the growth and spread of cancerous cells in several in vivo and in vitro studies [83,84,85,86,87]. This opens the possibility of designing therapeutic drug delivery systems specifically targeting the cancerous cells in the colon [86].

**Table 2:** A selection of explored health benefits and their action mechanisms.

Health Benefit	Action Mechanism	Pectin Source/Type	References
Reduced risk of gastric ulcers	Protection of the mucosal lining Normalizing gastric acid and pepsin levels Free radical scavenging activity.	Citrus/Apple	[87]
Lipid-lowering activity Cholesterol-lowering activity	Reduction in the bile acid reabsorption, Production of volatile fatty acids, Decrease in the cholesterol absorption.	Citrus	[88,89]
Hepatoprotective effects	Elimination of the toxic compounds via the intestine and the kidney, Antioxidant properties.	Citrus	[90,91]
Prebiotic activity (decreasing risk of diarrhea)	Modulating gut microbiome; facilitating colonic microbiotic balance.	Citrus/beet	[33,92,93]
Removal of toxic metal ions	Formation of the strong pectin-metal complexes; Elimination of the toxic metal ion.	Citrus/grapefruit	[94,95]
Reduced risk of colorectal cancer	Formation of the short-chain fatty acid as a result of the pectin bacterial fermentation.	Citrus	[93]
Inhibition of tumor growth	Inhibition of cell growth, reduction in cell attachment and chromatin fragmentation.	Citrus	[71]
Reduced metastasis rate	Suppression of the galectin-3-mediated agglutination.	Citrus	[96,97]

Pectin has been shown to exert a protective role on the gastric mucosa through multiple mechanisms. Its gel-forming capacity allows it to create a protective coating on the gastric lining, while simultaneously reducing gastric acid and pepsin secretion and exhibiting radical scavenging activity, thereby lowering the risk of gastric ulcer development [88,98]. Furthermore, pectin acts as a prebiotic substrate in the colon, where it is fermented by resident microbiota into short-chain fatty acids. This fermentation process normalizes gut bacterial balance and has been associated with a reduced incidence of diarrhea and improved overall gastrointestinal health [90,92].

Beyond its structural and delivery applications, pectin exhibits clinically relevant metabolic effects. Its lipid-lowering activity is attributed to the reduction in bile acid reabsorption in the intestine, stimulation of volatile fatty acid production, and decreased cholesterol absorption—collectively contributing to a favorable lipid profile [99]. Closely related are pectin’s hepatoprotective effects: by facilitating the elimination of toxic compounds via the intestine and kidneys, and through its intrinsic antioxidant properties, pectin has been shown to reduce hepatic oxidative stress and protect liver tissue from damage [33].

Pectin’s polyanionic character grants it a strong affinity for divalent and trivalent metal ions, enabling the formation of stable pectin-metal complexes. This property has been exploited for the removal of toxic metal ions from the gastrointestinal tract, representing a potential application in the management of heavy metal exposure [100].

### 4.1. Oral Administration Targeting Colon-Specific Delivery

Pectin has been extensively investigated as an excipient for oral controlled-release formulations, particularly for colon-targeted drug delivery. The main rationale lies in the fact that pectin resists degradation in the stomach and small intestine but is fermented by the colonic microbiota—a mechanism that can be exploited to selectively release drugs in the large intestine. Various natural polysaccharides have been tested for this purpose, but pectin holds a position among them [101].

For example, oral formulations, coated with a layer of pectin (often combined with insoluble polymers like ethylcellulose), might prevent the early dissolution of the drug in the upper gastrointestinal tract and ensure its release after exposure to the intestinal microbiota. High methoxylated pectin is mostly preferred for coating, because the high methoxilation reduces its solubility in stomach fluid and increases gelation [92,102].

A recent study showed the feasibility of using high-methoxylated pectin as a gastro-resistant coating film through different techniques (spray-coating vs. powder-layering), achieving delayed-release profiles and reproducible microbially triggered release, useful in the treatment of inflammatory bowel diseases [101].

Additionally, pectin-based matrix formulations (tablets or beads) have been proposed for colon-specific delivery; for example, pectin–zein microparticles have demonstrated the ability to protect active substances from digestive enzymes (with zein limiting particle swelling and porosity, and pectin protecting zein from proteases), ensuring the release of the active substance in the colon [103]. Such pectin-based colon-specific systems are being investigated for the administration of peptides, proteins, probiotics, and drugs intended for colon disorders (ulcerative colitis, colon cancer) [104,105]. In conclusion, pectin offers a flexible platform for targeted oral drug-delivery systems, as it can be processed into microspheres, gelatin capsules, films, or composites to meet various clinical requirements.

### 4.2. Transdermal Delivery Systems

The administration of active substances through the skin offers the advantage of avoiding hepatic metabolism and increasing patient compliance, as it is a non-invasive method. Pectin is used in transdermal patches in the form of hydrogels or adhesive matrices. The properties of pectin can be adjusted for transdermal applications by modifying its degree of esterification: highly methoxylated pectins are more hydrophobic, contributing to the prolonged release of hydrophobic compounds into the skin, whereas low-esterified (more hydrophilic) pectins can enhance the penetration of active substances through the skin [106,107].

A notable example is the use of pectin in dermal patch formulations for insulin delivery. Sibiya et al. showed that pectin–insulin patches (82.9 μg/kg) reduced blood glucose levels and improved diabetes-induced lipid abnormalities. The study concluded that pectin–insulin patches may protect against cardiovascular complications associated with diabetes and could represent a potential alternative to subcutaneous insulin therapy [108].

Beyond insulin, pectin is also being explored in transdermal patches for various drugs, where it serves as a gel-forming polymer that controls diffusion through the skin [109,110]. Pectin-based transdermal systems represent an emerging direction, offering a natural, biocompatible polymeric vector capable of delivering a wide range of therapeutic molecules across the skin barrier in a controlled manner.

### 4.3. Therapy and Diagnostics in Oncology

The applications of pectin in oncology follow two main directions: firstly, targeted drug delivery for anticancer agents, and secondly, its use as an adjuvant or diagnostic agent based on the intrinsic properties of pectin.

As a drug carrier, pectin can be formulated as nanoparticles, microspheres, or injectable hydrogels that deliver cytostatics directly to tumors, thereby reducing systemic side effects. For instance, pectin–magnetite or pectin–gold nanoparticles have been investigated as vehicles for doxorubicin and other cytotoxic agents, targeting preferential accumulation in tumors via the enhanced permeability and retention (EPR) effect or through surface ligand attachment.

A current trend involves the functional modification of pectin with tumor-targeting ligands: studies have reported folic acid-decorated pectin nanoparticles capable of binding to folate receptors overexpressed on cancer cells and selectively releasing drugs such as hydroxycamptothecin directly into tumor cells [111].

In parallel, modified pectins (usually known as Modified Citrus Pectins—MCPs) have attracted attention as antimetastatic and chemopreventive agents. MCP is a low-molecular-weight, low-esterified pectin derived from citrus pectin by controlled treatment; it can inhibit the protein galectin-3, which is implicated in tumor cell adhesion and metastasis [7]. Pectin is also thought to act by binding to immune receptors, including galectin-3, and thereby modulating cytokine release. Evidence suggests that pectins promote the production of anti-inflammatory cytokines, including IL-10 and TGF-β, while reducing levels of pro-inflammatory cytokines such as TNF-α and IL-6. This profile indicates that pectins may exert beneficial immunomodulatory effects rather than toxic effects within the organism [112,113].

Preclinical and clinical studies suggest that MCP can hinder the progression of certain cancers. For example, in a Phase II clinical trial involving patients with non-metastatic biochemically relapsed prostate cancer, oral administration of 4.8 g of MCP (PectaSol-C) three times daily resulted in disease stabilization in approximately 78% of patients after six months (46 out of 59 patients showed no progression), allowing entry into an extended follow-up phase [114].

Mechanistically, pectin (especially oligo-galacturonic fragments derived from its degradation) has been observed to induce apoptosis or sensitize cancer cells to chemotherapy, while also exhibiting beneficial immunomodulatory effects by stimulating anti-tumor cytokines [115,116].

In diagnostic applications, pectin-coordinated metallic nanoparticles are being explored as contrast agents in imaging. Thiolated pectin, for example, has been used to stabilize gold and iron oxide nanoparticles, producing biocompatible nanocomplexes (TPG-IN) that can be loaded with target molecules. These nanocomplexes have demonstrated potential for tumor imaging through enhanced contrast (e.g., in MRI) and even in improving tumor radiosensitivity during radiotherapy [117].

Lastly, pectin is used as a coating layer for silver or other metallic nanoparticles, generating antimicrobial nanosystems applicable in the management of oncological infections or local sterilization. For instance, silver nanoparticles coated with pectin have shown remarkable bactericidal activity against both Gram-positive and Gram-negative pathogens [75]. It has been proven that pectin provides oncology with both a safe and flexible delivery platform for drugs or contrast agents, as well as a promising adjuvant therapeutic agent owing to its documented anti-cancer bioactivities.

### 4.4. Pectin-Based Nanoparticulate Systems

The formulation of pectin into nanoparticulate systems has emerged as one of the most dynamic areas of pectin research, owing to the polymer’s intrinsic biocompatibility, ease of surface functionalization, and ability to form stable colloidal structures. Pectin-based nanoparticles can be prepared by a variety of methods, including ionic gelation, nanoprecipitation, and polyelectrolyte complexation, yielding particles in the 100–500 nm range suitable for parenteral, oral, or mucosal administration [118].

As drug carriers, pectin nanoparticles offer the advantage of protecting labile active substances from premature degradation while enabling controlled and targeted release. Pectin–magnetite and pectin–gold nanoparticles have been investigated as vehicles for cytotoxic agents such as doxorubicin, exploiting the enhanced permeability and retention effect for passive tumor accumulation [119]. Active targeting has been achieved by surface decoration with tumor-specific ligands: folic acid-functionalized pectin nanoparticles demonstrated selective binding to folate receptors overexpressed on cancer cells, enabling direct intracellular delivery of hydroxycamptothecin [120].

Beyond oncological applications, pectin nanoparticles have shown promise in antimicrobial therapy. Silver nanoparticles coated with pectin exhibit broad-spectrum bactericidal activity against both Gram-positive and Gram-negative pathogens, with the pectin shell improving colloidal stability and reducing cytotoxicity compared to uncoated silver nanoparticles [120,121]. This approach is particularly relevant in the context of wound infection management and oncological infection prophylaxis.

In diagnostic applications, pectin serves as a stabilizing matrix for metallic nanoparticles used as imaging contrast agents. Thiolated pectin has been employed to produce gold and iron oxide nanocomplexes (TPG-IN) with demonstrated utility in MRI contrast enhancement and tumor radiosensitization [122,123]. The multifunctional character of these systems—combining diagnostic and therapeutic functions in a single nanoplatform—positions pectin as a promising component of theranostic nanomedicine.

A further area of development involves pectin-based nanoparticles for oral protein and peptide delivery, where the polymer’s mucoadhesive properties and resistance to gastric degradation protect biologics until their release in the intestinal environment [124,125]. Taken together, pectin-loaded nanoparticulate systems bring forward new opportunities for research that combine drug delivery, diagnostics and antimicrobial therapy.

## 5. International Regulations on the Use of Pectin (EFSA, FDA, WHO)

### 5.1. In the European Union—European Food Safety Authority (EFSA)

Pectin (E 440i) and amidated pectin (E 440ii) are authorized as food additives, classified as thickeners, gelling agents, stabilizers, and emulsifiers. The European Food Safety Authority (EFSA) conducted a comprehensive re-evaluation of the safety of these additives in 2017, concluding that there is no safety concern regarding the use of pectin and amidated pectin in foods for the general population [7].

For adults and older children, no numerical Acceptable Daily Intake (ADI) has been established, as pectin is considered safe within the limits of good manufacturing practice (ADI “not specified”). However, certain sensitive population groups required special attention: in 2021, the EFSA Panel on Food Additives and Flavorings (FAF) evaluated the use of pectin in infant formulas (<16 weeks). It was found that at the previously permitted maximum level (0.5% in infant formula), pectin intake could lead to excessive methanol exposure (derived from pectin de-esterification), exceeding the safety margin for infants [126].

As a result, EFSA recommended lowering the maximum allowed pectin concentration in follow-on formulas for infants to 0.2%, to prevent any potential adverse effects (e.g., methanol accumulation or laxative effects). Additionally, EFSA proposed stricter purity specifications for pectins, introducing tighter limits for contaminants such as arsenic, lead, cadmium, mercury, and aluminum, as well as microbiological criteria, thereby ensuring that industrially used pectin maintains a high degree of purity and safety [126].

At the European level, pectin (including amidated pectin) is recognized as safe, with special precautions applying only to infant use, and is regulated in the Codex Alimentarius as additive E440 without the need for a numerical ADI (Acceptable Daily Intake).

### 5.2. In the United States—Food and Drug Administration (FDA)

The U.S. Food and Drug Administration (FDA) includes pectin on the list of GRAS (Generally Recognized as Safe) substances for direct use in food. According to 21 CFR §184.1588, pectins (including those with different degrees of esterification, amidated pectin, pectinates, and pectic acids) may be added to foods without quantitative limitation, provided that good manufacturing practice (GMP) is observed [75].

Pectin is approved as an emulsifying, stabilizing, and gelling agent in numerous food products, ranging from jams and jellies to soluble fiber supplements. It is also listed in the United States Pharmacopeia (USP) as a pharmaceutical excipient—for instance, as a binder in tablets and as a release-retarding agent in certain oral formulations.

The FDA does not impose a maximum dose, considering dietary intake of pectin to be safe and self-limiting due to gastrointestinal tolerance (high doses may cause bloating or a laxative effect, but no significant systemic toxicity). In summary, under U.S. regulation, pectin enjoys a favorable status as a long-established, well-characterized ingredient approved both as a food additive and a pharmaceutical excipient, with no strict dosage restrictions if GMP standards are maintained [NO_PRINTED_FORM] [75].

### 5.3. World Health Organization (WHO)—Joint Food and Agriculture Organization (FAO)/WHO Expert Committee on Food Additives (JECFA)

The Joint FAO/WHO Expert Committee on Food Additives (JECFA) has evaluated pectins on multiple occasions.

According to JECFA’s report, pectins (amidated and non-amidated) are normal constituents of the human diet and have even been administered intravenously in high doses to human volunteers without notable acute toxic effects. Toxicological studies in animals support this safety profile: dietary supplementation with pectin up to 5% in rats produced no adverse effects (only a compensatory enlargement of the cecum due to fiber fermentation). Even at 10% dietary inclusion, amidated pectin caused only a minor reduction in growth rate in rats—a non-significant effect; two-year studies showed no meaningful toxicological differences between amidated and non-amidated pectin [127].

JECFA has also specifically examined pectin use in infants (similarly to EFSA) and, based on a study in newborn piglets, concluded that adding 0.2% pectin to infant formula is safe, with a sufficient margin of exposure relative to the no-adverse-effect level (MOE~2.4–2.9) [51]. At 0.5%, however, infant exposure approached levels where minor growth effects were observed; thus, 0.5% was considered too high [75,127].

In conclusion, the WHO/JECFA classifies pectin among the safest food additives, permitting unrestricted use in general foods and recommending caution only for formulations intended for very young infants.

### 5.4. Other International Significant Regulatory Framework

There are several national and regional authorities that have independently regulated the use of pectin, considering its safety profile, and the consensus remains broadly consistent throughout. Considering pectin’s extensive global history of use as a food additive and lately a pharmaceutical excipient, this polysaccharide is accepted as safe for use.

China is one of the largest producers and consumers of pectin and, therefore, has specifically addressed the matter of pectin safety. Its regulatory body, the National Medical Products Administration (NMPA), permits the use of pectin under the national standard GB 2760, which governs the use of food additives in the People’s Republic of China [128].

Australia and New Zealand’s joint regulatory body aligns with EFSA and JECFA decisions through the Food Standard 1.3.1 [129], where it is stated that the use of pectin is permitted as a food additive without a maximum level. Food Standards Australia New Zealand (FSANZ) regulation is incumbent on the need for good manufacturing practices to be observed [129].

Brazil’s prominent position as a producer of tropical fruit and the opportunity of increased pectin extraction from the by-product—specifically mango and passion fruit peels—has urged its regulatory authority, the National Health Surveillance Agency (ANVISA), to give a resolution on pectin. Resolution RDC No. 380/2025 permits the use of pectin as a food additive and technological adjuvant, essentially harmonizing national regulations with international ones [130].

Canada’s authority on food safety, Health Canada, aligned with the FDA by classifying pectin as an approved food additive under the Food and Drug Regulations (FDR), permitted for use as a gelling agent, stabilizer and thickener [131].

The regulatory consensus on pectin reinforces the safety profile as both a food additive and a pharmaceutical excipient. This facilitates global trade and encourages study on pectin-based applications while also supporting its use in emerging biomedical research.

## 6. Toxicity and Safety of Pectin and Its Derivatives in the Biomedical Context

### 6.1. General Toxicological Profile

Pectin is recognized as a non-toxic polymer, well-tolerated by the human body. Being essentially a soluble fiber, high doses of ingested pectin can cause gastrointestinal discomfort (bloating, mechanical laxative effect), but no systemic toxic effects. Animal studies have shown no toxicity even at extremely high intake levels: diets containing 5–10% pectin did not cause organ damage or hematological alterations, except for minor physiological adaptations (e.g., increased cecum weight due to fiber fermentation) [132].

Pectin’s major biomedical advantage lies in its biocompatibility—its favorable interaction with tissues and cells. Pectin-based hydrogels and surfaces do not induce acute inflammatory responses or cytotoxicity. On the contrary, pectin has been observed to exert local anti-inflammatory effects: for example, in vivo studies have shown reduced systemic and intestinal inflammation in animals treated with low-esterified pectin [133].

The proposed mechanism involves pectin binding to immune receptors (e.g., lectin-type receptors, such as galectin-3 mentioned earlier) and modulating cytokine release. Pectins appear to stimulate the production of anti-inflammatory cytokines (IL-10, TGF-β) while decreasing pro-inflammatory ones (TNF-α, IL-6), giving them a beneficial rather than toxic profile within the organism [134,135].

Pectin exerts its immunomodulatory effects through both direct and indirect mechanisms. Among the direct effects, pectin interacts with immune cells through electrostatic interactions with dendritic cells and macrophages, leading to the blockage of pro-inflammatory Toll-like receptor TLR2/1 and TLR4 signaling pathways—an effect particularly associated with citrus pectin fractions with lower degrees of methylation. Additionally, highly esterified degree pectin has been shown to inhibit macrophage activation by suppressing iNOS, COX-2, IKK, NF-κB and MAPK signaling, resulting in reduced pro-inflammatory activity. Pectin also interacts with G-protein coupled receptors (GPR), contributing to a protective effect on epithelial barrier function, and modified low molecular weight pectin has been shown to activate T cells, B cells and natural killer cells, stimulating adaptive immunity [112,113].

### 6.2. Safety of Pectin Derivatives

In many biomedical applications, pectin is chemically modified (e.g., amidation, oxidation, sulfhydrylation) to improve its functional properties. These modifications raise safety questions, particularly due to native pectin’s non-toxic character. Available data suggest that amidated pectin—the only derivative authorized for food use—has a safety profile very similar to that of native pectin. Chronic studies in rats fed 10% amidated pectin showed only a slight reduction in growth, without other signs of toxicity or teratogenic effects; at 2–5% dietary inclusion, no differences from non-amidated pectin were observed [7].

Thus, amidated pectin is considered safe for consumption and medical use, with EFSA identifying no specific safety concerns for this [7]. Pectins with different degrees of methoxylation (high-methoxylated vs. low-methoxylated) are metabolized similarly by the colonic microbiota; however, highly methylated pectins release more methanol upon degradation. In adults, the amount of methanol generated and absorbed from typical pectin doses is negligible compared to endogenous production or fruit intake. In very young infants (under 3 months), however, methanol metabolism is less efficient, which explains the precautionary limits on pectin in infant formulas [126].

From a pharmaceutical standpoint, the safety of pectin and its derivatives has been demonstrated in multiple preclinical studies: for instance, pectin-based wound dressings cause no skin irritation or allergic reactions; a new pectin–honey hydrogel tested in animal wound models accelerated healing without notable adverse effects [136]. Similarly, pectin-based tissue implants (e.g., pectin–collagen hydrogels for bone engineering) have proven non-immunogenic and gradually resorbed without chronic fibrosis [137].

### 6.3. Tolerance and Compatibility Considerations

In vivo, pectin is generally degraded into fermentable oligomers by the intestinal microbiome or by macrophages (which can secrete acidic hydrolases). The degradation products—mainly galacturonic acid and oligosaccharides—are harmless. Moreover, pectins have been studied as prebiotics, stimulating the growth of beneficial colonic microbiota (e.g., Bifidobacteria) and improving gastrointestinal health [138].

Biocompatibility is also evident at the cellular level: in vitro experiments show that mammalian cells (fibroblasts, keratinocytes, osteoblasts) can adhere to and proliferate on pectin-treated or pectin–composite surfaces without cytotoxicity or oxidative stress [137]. A clear example is the use of pectin in 3D cultures of mesenchymal stem cells for cartilage regeneration: photo-crosslinked methacrylated pectin formed porous scaffolds supporting chondrogenic differentiation, and after implantation in animals, they were colonized by cells without acute immune reactions. In implantable devices, enzymatic degradation of pectin can be slower (in the absence of microbiota), but it can be tuned by modifications (e.g., introducing lysozyme-sensitive linkages or locally adding pectinolytic enzymes) [139].

## 7. Recent Trends in the Biocompatibility, Degradability, Functional Modifications, and Multidisciplinary Applications of Pectin

### 7.1. Biocompatibility and Biological Interactions

Current research trends in pectin focus on optimizing its interaction with biological systems. Pectin is already recognized as non-toxic and biocompatible, but recent studies investigate ways to enhance its bio-interactivity—for example, by functionalizing pectin with bioactive peptide sequences (such as RGD or others) to improve cell adhesion and proliferation on scaffolds in tissue engineering. “Smart” pectins that respond to biological stimuli are also being explored, such as enzyme-sensitive pectin hydrogels (which degrade faster in the presence of specific enzymes from target tissues) or pH-responsive gels (which swell at the pathological tissue’s pH, thereby enabling targeted drug release) [5,140].

Another emerging area involves the use of pectin in artificial microbiomes: encapsulating probiotic bacteria in pectin microgels to protect them from harsh conditions and deliver them to the intestine. These systems serve dual roles—pharmaceutical (microbiota restoration) and material (pectin as a probiotic biomaterial) [141].

The long-term biocompatibility of pectin-based implants is also being investigated. Current results indicate an absence of chronic toxicity, though subtle tissue responses (e.g., fibrosis at the implantation site) are being evaluated in comparison with other biomaterials) [137]. Overall, pectin continues to stand out as a cell-friendly biomaterial, with recent research strengthening this position and expanding its applicability into new clinical directions.

### 7.2. Degradability and Sustainability

An important trend is the integration of pectin into the concept of “eco-pharmacy” and sustainable materials. Being naturally biodegradable (by composting or environmental microbiota), pectin leaves a minimal ecological footprint after use. This feature is leveraged in two main ways: (i) biodegradable medical devices—for instance, drug delivery systems that decompose in the body after releasing the therapeutic agent, thus eliminating the need for surgical removal (e.g., oesophageal or ureteral stents made of pectin that gradually dissolve post-therapy)and (ii) reduction in pharmaceutical pollution—pectins are proposed as “green” excipients that could replace poorly biodegradable synthetic polymers (like HPMC or PVA) in various formulations, thereby reducing the environmental impact of pharmaceutical waste [142,143].

Progress is also being made in developing more eco-friendly pectin extraction methods (e.g., ultrasound-assisted extraction, supercritical fluids, or natural deep eutectic solvents), making the entire life cycle of pectin—from production to disposal—sustainable [15,48,50,72].

Controlled degradability is another research topic: by adjusting the degree of esterification or introducing labile linkages (e.g., enzyme-cleavable bonds), pectins with specific degradation times (from days to months) can be engineered, tailored to their intended application (e.g., wound healing versus long-term tissue scaffolding) [144,145].

Although pectin offers numerous benefits, it also has several limitations that should be taken into account in future research. Pectin forms hydrogels with relatively weak mechanical strength compared to synthetic polymers, which restricts its application in load-bearing contexts unless it is reinforced or chemically crosslinked [77]. Variability between batches of commercially available pectin, due to variations in plant sources, extraction techniques, and degrees of esterification, can also impact reproducibility during formulation [146]. Also, developing pectin-based nanoparticle systems on an industrial scale could prove difficult, as does achieving accurate control over their in vivo degradation behavior [147]. Overcoming these challenges will require standardized extraction methods, the design of hybrid composite materials, and more extensive in vivo and clinical investigations to achieve pectin’s potential as a biomedical material.

### 7.3. Innovative Functional Modifications

From a polymer chemistry perspective, pectin research has seen remarkable growth in recent years, with numerous new derivatives and modifications reported. The main goal is to endow pectin with novel functionalities, moving beyond its traditional role as an inert gelling agent. The structural customization of pectin for each specific application, through targeted chemical modifications, focuses on improving performance—whether in drug loading capacity, targeting specificity, mechanical strength, or biological activity.

One direction aims to enhance pectin’s interaction with bioactive molecules—for example, acylation with gallic acid or other phenolic acids yields amphiphilic derivatives that can solubilize hydrophobic compounds while exhibiting intrinsic antioxidant and antibacterial properties. Such acylated pectins have been tested as high-performance pharmaceutical emulsifiers and as edible films with prolonged antimicrobial action [148,149].

Another approach involves grafting polymers or reactive groups onto the pectin backbone. For instance, methacrylated pectin (Pec-MA)—obtained by substituting hydroxyl groups with vinyl methacrylate—can be crosslinked under UV light, enabling the rapid formation of injectable hydrogels directly in the body (using a UV or LED lamp in minimally invasive procedures). Pec-MA has demonstrated biocompatibility and, as noted, showed enhanced antitumour activity in vitro compared to native pectin (e.g., inhibition of colon cancer cell proliferation), paving the way for its in vivo evaluation as a chemo-embolizing agent (a hydrogel that locally releases cytostatic while blocking tumor vascularization) [107,150].

Cationic pectins represent another trend: by introducing amino or quaternary groups, the anionic pectin is transformed into a cationic polyelectrolyte capable of forming complexes with nucleic acids (DNA/RNA). These cationic pectin derivatives are being studied as non-viral vectors for gene therapy, promising better biocompatibility than synthetic polymers (e.g., PEI) and enzymatic degradation after gene release. The cationic groups also impart excellent mucoadhesive properties (through interactions with negatively charged mucins)—as reported in earlier studies, pectins bearing primary amine groups showed superior mucosal adhesion, making them effective for nasal and other mucosal delivery routes [151,152].

Another line of research involves the conjugation of biological ligands—for example, terpyridine-modified pectins that can complex with metal ions, granting the material enhanced antibacterial activity via controlled metal ion release [153].

### 7.4. Multidisciplinary Applications and Domain Convergence

A remarkable feature of pectin is its presence at the intersection of multiple disciplines: food technology, pharmacology, materials engineering, nanotechnology, and clinical medicine. The most recent trends highlight precisely this multidisciplinary convergence.

In the global context of replacing conventional plastics and developing sustainable medical materials, pectin has emerged as a key candidate for biodegradable bioplastics and interdisciplinary applications. Due to its hydrophilic polysaccharide nature, pectin can be processed into films, fibers, or foams with performance properties suitable for various uses.

For instance, in active and intelligent food packaging, pectin is employed to create edible and biodegradable films, sometimes loaded with antimicrobial agents or freshness indicators [154]. This trend of using pectin as a packaging biopolymer reduces dependence on fossil-based plastics and provides properties such as oxygen barrier capacity and the ability to retain useful volatile compounds [155].

In medicine, pectin-based bioplastics are being developed for temporary medical devices (for example, tissue regeneration matrices that degrade after healing) or for resorbable prosthetic components. A concrete example of a biomaterial already in clinical use is the hydrocolloid dressing: these gelling patches, used for the treatment of chronic wounds (ulcers, pressure sores), contain pectin combined with carboxymethylcellulose and gelatine [156,157]. The pectin in these dressings absorbs exudate and forms a soft gel at the wound surface, maintaining an optimal moist environment for healing while also exhibiting a local anti-inflammatory effect. Such hydrocolloids demonstrate the biocompatibility of pectin in direct contact with tissues and their capacity to accelerate epithelialization. Other emerging applications include the use of pectin in bio-inks for 3D tissue printing, in ocular delivery devices (ophthalmic films that dissolve on the surface of the eye), or as a nucleation agent for crystal growth in the manufacture of implantable drug delivery systems [156,158].

Due to its chemical versatility, pectin can be combined with numerous other natural polymers (cellulose, alginates, chitin, etc.) or with inorganic nanoparticles, generating intelligent composites. For example, pectin has been incorporated into conductive hydrogels (containing carbon nanotubes) for electrically stimulated controlled drug release or crosslinked with ferric vanadyl chloride to obtain self-healing materials suitable for advanced wound dressings [159,160].

In the cosmetic and dermatological sectors, pectin is incorporated into hydrogels for facial masks that release anti-aging actives, technologies closely related to those used in transdermal pharmaceutical systems [160]. Another emerging trend involves combining pectin with advanced technologies such as 3D printing and microfluidics: pectin microcapsules produced via microfluidic systems offer precisely controlled sizes for individualized drug doses, while 3D bioprinting with pectin–cellulose bioinks produces porous scaffolds for customized cell cultures [150,156,161].

The use of pectin has expanded considerably in recent years, encompassing applications such as edible food-protective coatings, antimicrobial bio-derived films, nanoparticulate delivery systems, wound-healing materials, and cancer treatment platforms. Innovations in extraction technologies, diversification of botanical sources, and enhanced understanding of structural modification strategies have substantially improved its physicochemical properties, recovery yields, and technological versatility [72].

## 8. Conclusions

Eco-pharmacy is a particular area of pharmacological studies that aims at reducing the polluting effects of pharmaceutical products on the environment, and an important part of that is focusing on environmentally friendly substances that degrade without causing harm in nature. Pectins are a remarkable group of hydrocolloids, and even if they have been adopted from the food sector, they are an important part of developing a mindful use of resources in the pharmaceutical and medical industries.

An ongoing search for new extraction and disposal methods with a reduced impact on the environment makes natural-sourced biomaterials a valuable resource to be continually explored. Pectin has evolved beyond its traditional role as a food additive, becoming a central player in the field of modern biomaterials.

This synthesis has shown that pectin and its derivatives offer promising platforms for oral and transdermal drug delivery, make unique contributions to oncology (as drug carriers and anti-metastatic agents), provide eco-friendly solutions for developing medical bioplastics and biodegradable packaging, and exhibit an excellent safety profile confirmed by major regulatory authorities (EFSA, FDA, WHO). Its ability to undergo chemical modification gives pectin exceptional flexibility to meet diverse functional requirements—from enhanced mucoadhesion to stimuli-responsive properties and molecular targeting. Although pectin, as a biomaterial, offers many directions of study, it is also appropriate to mention various limitations. As a standalone hydrogel, its mechanical strength is lower than that of synthetic polymers, which restricts its use in applications requiring structural support. Secondly, the variability between commercial pectin batches, which arises from differences in plant source, extraction method and degree of esterification, can affect the reproducibility of formulations. Future research should focus on standardizing extraction processes, developing pectin-based composite materials, and conducting more in-depth in vivo studies to address these limitations.

Recent trends indicate an increasing multidisciplinary character in pectin research, integrating insights from materials science, biology, and food science to create the next generation of intelligent and sustainable therapeutic systems. With such a portfolio of attributes—biocompatibility, biodegradability, safety, and versatility—pectin stands out as a key natural polymer for the development of future biomedical innovations.

## Data Availability

No new data were created or analyzed in this study. Data sharing is not applicable to this article.
